# Heteroatom and solvent effects on molecular properties of formaldehyde and thioformaldehyde symmetrically disubstituted with heterocyclic groups C_4_H_3_Y (where Y = O–Po)

**DOI:** 10.1007/s00894-017-3435-4

**Published:** 2017-08-21

**Authors:** Piotr Matczak, Małgorzata Domagała

**Affiliations:** Department of Theoretical and Structural Chemistry, Faculty of Chemistry, University of Łódź, Pomorska 163/165, 90-236 Lodz, Poland

**Keywords:** Quantum chemical calculations, Molecular properties, Five-membered heterocyclic substituents, Group 16 heteroatoms

## Abstract

In this work several molecular properties of symmetrically disubstituted formaldehyde and thioformaldehyde have been studied using a quantum chemistry approach based on density functional theory. Five-membered heteroaromatic rings containing a single group 16 heteroatom were taken into account as the substituents (i.e., furan-2-yl, thiophen-2-yl, selenophen-2-yl, tellurophen-2-yl, and the experimentally as yet unknown polonophen-2-yl). For the resulting ten formaldehyde and thioformaldehyde derivatives, the geometry, energetics, frontier molecular orbitals, dipole moment and polarizability of their molecules were examined in order to establish the effect of ring heteroatom on these properties. Furthermore, these properties were also determined for the molecules in three solvents of low polarity (benzene, chloroform, and dichloromethane) in order to expand our study to include solvent effects. The dipole moment and polarizability of the investigated molecules show regular variations when the ring heteroatom descends through group 16 and the solvent polarity grows. The heteroatom and/or solvent effects on the part of the studied properties are, however, more complex. An attempt is made to rationalize the observed variations in the molecular properties. The conformational behavior of the investigated molecules was also explored and the conformationally weighted values of dipole moment and polarizability are presented.

**Electronic supplementary material:**

The online version of this article (doi:10.1007/s00894-017-3435-4) contains supplementary material, which is available to authorized users.

**Graphical abstract:**
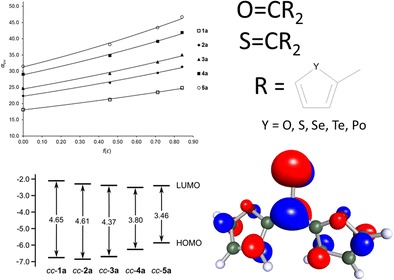
Some molecular properties of symmetrically disubstituted formaldehyde and thioformaldehyde

## Introduction

Organic molecules containing heterocyclic fragments constitute one of the most important research areas in modern organic, bioorganic and medicinal chemistry [1–3]. The importance of such molecules is reflected in their innumerable applications, at both laboratory and industrial scale. A reason for this importance arises from the possibility of modifying existing functions or imparting new desired features to organic molecules through the introduction of one or more heteroatoms into their cyclic fragments. The presence of heteroatoms in the resulting heterocyclic fragments leads to the redistribution of electron density and changes in possible aromatic character [4], which allows for the manipulation of various molecular properties, such as reactivity, optoelectronic properties, chelating ability and many others. These properties may be strongly affected by the occurrence of a heteroatom in a heterocyclic fragment, and by the kind of heteroatom introduced. This is usually referred to as the heteroatom effect. In the case of molecules with substituents being five-membered aromatic monocycles with a single group 16 heteroatom (furan, thiophene, selenophene, tellurophene), it is known that the heteroatom effect is responsible for changes in their properties that respond to many demands relevant to organic synthesis [5–7], optics [8–10], electronics [11], material science [12], pharmacology [13] and biology [14]. If such molecules are in solution, the surrounding solvent usually exerts an additional influence on their properties. For instance, the dependence of their reactivity [6, 15], conformational preference [16–18], reaction kinetics [19] or spectroscopic properties [20, 21] on the presence of a solvent and its polarity has been detected.

In this work, a set of ten molecules representing symmetrically disubstituted formaldehyde and thioformaldehyde is the subject of a quantum chemical investigation of their molecular properties. Five-membered heterocyclic substituents C_4_H_3_Y containing a single group 16 heteroatom Y (furan-2-yl for Y = O, thiophen-2-yl for Y = S, selenophen-2-yl for Y = Se, tellurophen-2-yl for Y = Te, and the experimentally as yet unknown polonophen-2-yl for Y = Po) are taken into account. The resulting two series of formaldehyde and thioformaldehyde derivatives, also termed here symmetrical diheteroaryl ketones and thioketones, are interesting from both theoretical and experimental points of view. In general, they are useful for the synthesis of other important organic compounds [7, 22, 23]. The preparative perspective also reveals their mutual connection. Such thioketones can be prepared from corresponding ketones via oxygen/sulfur exchange, as has very recently been carried out using Lawesson’s reagent [24]. Here, the geometry, energetics, frontier molecular orbitals, dipole moment and polarizability in vacuum and in solvents, were obtained for the aforementioned two series of molecules, using a density functional theory method and an implicit solvation model. Tracking variations in these properties along the two series of molecules allowed us to establish how strongly these properties are linked with the kind of ring heteroatom and the presence of solvent. To the best of our knowledge, there are only sparse computational studies that include an analysis of heteroatom and/or solvent effects for some symmetrical diheteroaryl ketones and thioketones [17, 25, 26]. In the case of the title compounds, only molecules with light ring heteroatoms (most often with sulfur) have been studied using quantum chemical methods so far [17, 20, 21, 26–28]. This work extends our previous computational study of symmetrical diheteroaryl ketones and thioketones [26] to the full range of group 16 ring heteroatoms, and, additionally, it includes the effect of solvent.

## Computational details

**Scheme 1:**
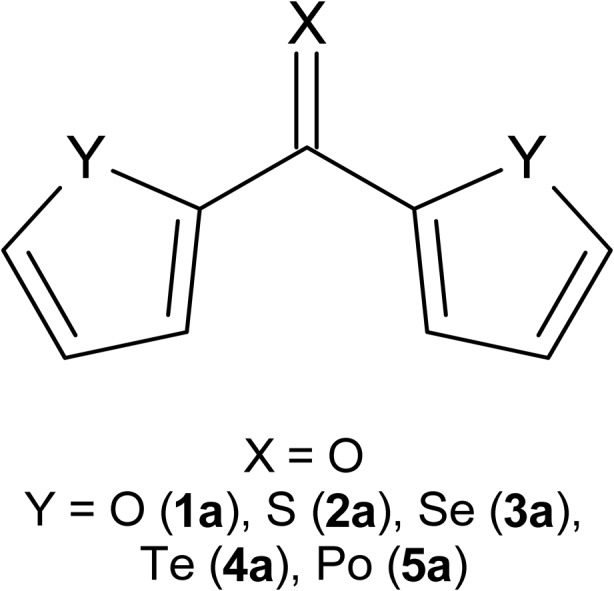
Symmetrically disubstituted formaldehyde **1a**–**5a**

The molecular geometries of five symmetrical diheteroaryl ketones (**1a**–**5a**, see Scheme 1) and their thiocarbonyl analogs (**1b**–**5b**) were optimized in vacuum at the RI-B3LYP/def2-QZVPP level of theory [29–33]. The obtained geometries of isolated molecules were subsequently optimized in the presence of a solvent, using RI-B3LYP/def2-QZVPP and the COSMO implicit solvation model [34]. Three solvents of low polarity, in terms of both their dipole moment and dielectric constant, were considered. In order of increasing polarity, these solvents are benzene, chloroform, and dichloromethane. Our interest in solvents of low polarity arises from their frequent use in the synthesis and reactions of diheteroaryl ketones and thioketones [24]. Harmonic vibrational frequency calculations yield no imaginary frequencies for all the optimized molecular geometries. For the molecules containing heavy atoms, such as Te in **4a** and **4b**, and Po in **5a** and **5b**, the inner electrons of these atoms are modeled by the Stuttgart effective core potentials [35, 36] provided with the def2-QZVPP basis set. The [Ar]3*d* ^10^ inner electrons of Te and the [Kr]4*d* ^10^4*f* ^14^ inner electrons of Po are replaced with the Stuttgart effective potentials. The application of these effective core potentials allows for the inclusion of scalar relativistic effects in the geometry optimizations of **4a**, **4b**, **5a**, and **5b**. Such a one-component treatment of relativity should be sufficient for obtaining molecular geometries with reasonable accuracy, because scalar relativistic effects are usually much more important for geometries than spin-orbit relativistic effects [37, 38]. However, an accurate determination of energetics for molecules with heavy atoms requires a computational treatment accounting for spin-orbit coupling. Therefore, the final total energies of **4a**, **4b**, **5a**, and **5b** have been recalculated at the two-component relativistic RI-B3LYP/dhf-QZVPP-2c level of theory [39, 40]. The sizes of Te and Po cores described by the dhf-QZVPP-2c effective potentials are the same as those defined by def2-QZVPP. The calculations of molecular dipole moments, average static dipole polarizabilities, and polarizability anisotropies were carried out using the aforementioned levels of theory. In addition, the def2-QZVPP and dhf-QZVPP-2c basis sets were augmented with a small number of moderately diffuse functions recommended for efficient computations of electronic response properties [41]. Test calculations performed for a set of small molecules with structural features occurring in **1a**–**5a** and **1b**–**5b** confirm that the use of these diffuse functions does improve the accuracy of the predicted molecular dipole moments and polarizabilities (see sections S1 and S2 in the Electronic Supplementary Material).

The selection of B3LYP for this work was originally based on the well-known good performance of this density functional in predicting geometries and energies [42]. The accurate prediction of electric properties is generally a more difficult challenge for B3LYP, with its performance then depending on which electric property is calculated and what class of molecules is considered [43, 44]. There are several benchmark studies that report a relatively good performance of B3LYP for calculating low-order ground-state electric properties such as dipole moment and polarizability [45–47]. The results of our test calculations from section S2 also indicate the satisfactory performance of B3LYP for molecular dipole moments and polarizabilities in a set of small molecules with structural features occurring in **1a**–**5a** and **1b**–**5b**.

All calculations were carried out using TURBOMOLE (versions 6.5 and 7.0.1) [48]. Contours of frontier molecular orbitals were visualized using TmoleX 4.1 [49].

## Results and discussion

### Molecular geometry and stability

**Scheme 2:**
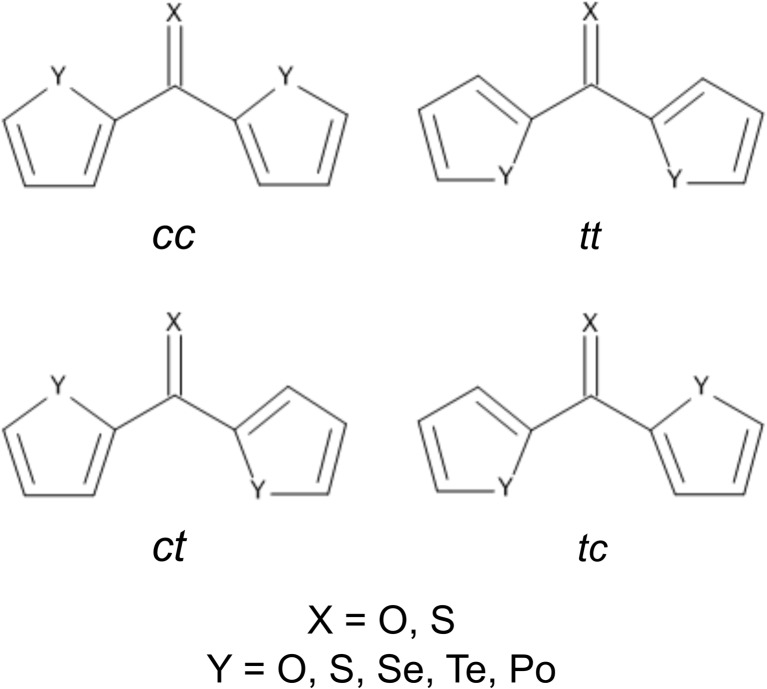
Schematic representation of possible conformations for molecules **1a**–**5a** and **1b**–**5b**

The molecules of the investigated compounds can adopt three conformations, differing in the orientation of ring heteroatoms (Y = O–Po) relative to the carbonyl oxygen atom (X = O) in **1a**–**5a** or the thiocarbonyl sulfur atom (X = S) in **1b**–**5b**. The first type of conformation exists if X and both Y atoms are on the same side of the C–C bonds linking the heteroaryl substituents with the C=X group. In other words, both Y atoms display a cis-like orientation with respect to the X atom. The resulting conformer will be denoted here by the prefix *cc*. The trans-like orientation of X relative to Y of both heteroaryl rings leads to the *tt*-conformer. Finally, the third type of conformation combines a X,Y-cis-like orientation for one heteroaryl ring and a X,Y-trans-like orientation for another ring. The *ct*- and *tc*-conformers are regarded to be identical due to disubstitution with the same heteroaryl group. In other words, the third type of conformation refers to two degenerate structures (a schematic visualization of possible conformations is presented in Scheme 2). Torsion angles *τ* formed by three consecutive bonds in the X=C–C–Y fragments are suitable geometrical parameters for characterizing the three conformations. Due to its symmetrical molecular geometry, each *cc*- and *tt*-conformer possesses a pair of identical *τ* twist angles. The values of *τ* for all conformers of **1a**–**5a** and **1b**–**5b** are shown in Tables 1 and 2.

**Table 1:** Twist angles between the X and Y atoms of the X=C–C–Y fragments (*τ* in °) and relative Gibbs free energies (Δ*G* in kcal mol^−1^) for the conformers of **1a**–**5a** in vacuum and solvents

Conformer	Medium							
	Vacuum		Benzene		Chloroform		Dichloromethane	
	*τ*	Δ*G* ^a^	*τ*	Δ*G* ^a^	*τ*	Δ*G* ^a^	*τ*	Δ*G* ^a^
*cc* **-1a**	−17.7	2.84 [0.3]	−17.0	2.17 [1.1]	−16.5	1.76 [2.3]	−16.1	1.43 [4.0]
*ct* **-1a**	0.0 (179.9)	0.00 [80.7]	0.0 (179.9)	0.00 [87.8]	0.0 (179.9)	0.00 [88.8]	0.0 (179.9)	0.00 [89.0]
*tt* **-1a**	180.0	0.45 [19.0]	180.0	0.81 [11.1]	180.0	0.95 [9.0]	180.0	1.09 [7.0]
*cc* **-2a**	−18.6	0.00 [38.8]	−18.5	0.00 [44.1]	−18.4	0.00 [49.7]	−18.2	0.00 [53.2]
*ct* **-2a**	−15.9 (157.9)	0.17 [58.4]	−16.6 (157.2)	0.30 [53.3]	−16.9 (156.9)	0.43 [48.2]	−17.1 (156.7)	0.51 [44.9]
*tt* **-2a**	159.2	1.56 [2.8]	158.0	1.67 [2.6]	157.4	1.85 [2.2]	157.2	2.00 [1.8]
*cc* **-3a**	−18.1	0.00 [66.7]	−18.7	0.00 [68.8]	−19.0	0.00 [68.8]	−19.1	0.00 [71.3]
*ct* **-3a**	−16.1 (154.6)	0.84 [32.6]	−16.8 (154.0)	0.89 [30.5]	−17.2 (153.7)	0.89 [30.5]	−17.4 (153.6)	0.96 [28.0]
*tt* **-3a**	155.9	2.73 [0.7]	154.9	2.70 [0.7]	154.3	2.73 [0.7]	154.1	2.79 [0.6]
*cc* **-4a**	−17.0	0.00 [90.3]	−17.5	0.00 [87.7]	−17.8	0.00 [86.3]	−18.0	0.00 [85.2]
*ct* **-4a**	−15.3 (150.4)	1.74 [9.6]	−16.3 (149.8)	1.58 [12.1]	−16.9 (149.5)	1.51 [13.5]	−17.3 (149.5)	1.46 [14.5]
*tt* **-4a**	152.4	4.16 [0.1]	151.1	3.82 [0.1]	150.5	3.58 [0.2]	150.2	3.46 [0.2]
*cc* **-5a**	−16.7	0.00 [95.2]	−17.8	0.00 [93.9]	−18.5	0.00 [92.3]	−19.0	0.00 [90.8]
*ct* **-5a**	−14.8 (148.6)	2.19 [4.7]	−15.7 (147.8)	2.03 [6.1]	−16.4 (147.4)	1.89 [7.6]	−16.9 (147.3)	1.78 [9.0]
*tt* **-5a**	151.1	4.80 [0.0]	149.7	4.34 [0.1]	149.2	4.01 [0.1]	148.9	3.77 [0.2]

^a^The percentage abundances of individual conformers in the equilibrium mixture of three conformers of each compound are shown in square brackets

**Table 2:** Twist angles between the X and Y atoms of the X=C–C–Y fragments (*τ* in °) and relative Gibbs free energies (Δ*G* in kcal mol^−1^) for the conformers of **1b**–**5b** in vacuum and solvents

Conformer	Medium							
	Vacuum		Benzene		Chloroform		Dichloromethane	
	*τ*	Δ*G* ^a^	*τ*	Δ*G* ^a^	*τ*	Δ*G* ^a^	*τ*	Δ*G* ^a^
*cc* **-1b**	−21.4	2.58 [0.6]	−21.0	1.70 [2.5]	−20.6	1.27 [5.2]	−20.2	0.99 [8.2]
*ct* **-1b**	−6.4 (172.5)	0.00 [89.9]	−7.8 (170.4)	0.00 [87.2]	−8.4 (169.5)	0.00 [88.0]	−8.5 (169.3)	0.00 [86.9]
*tt* **-1b**	164.5	0.92 [9.5]	164.5	0.85 [10.3]	164.8	1.10 [6.9]	165.0	1.29 [4.9]
*cc* **-2b**	−22.8	0.00 [49.3]	−22.8	0.00 [53.1]	−22.7	0.00 [56.3]	−22.7	0.00 [58.5]
*ct* **-2b**	−21.5 (149.7)	0.42 [48.8]	−21.9 (149.6)	0.51 [45.1]	−22.2 (149.7)	0.58 [42.1]	−22.3 (149.9)	0.63 [40.1]
*tt* **-2b**	150.6	1.92 [1.9]	150.2	2.00 [1.8]	150.1	2.11 [1.6]	150.1	2.21 [1.4]
*cc* **-3b**	−22.2	0.00 [67.4]	−22.5	0.00 [67.6]	−22.6	0.00 [68.4]	−22.6	0.00 [70.2]
*ct* **-3b**	−19.9 (146.3)	0.85 [31.9]	−20.7 (146.5)	0.86 [31.5]	−21.2 (146.7)	0.88 [30.7]	−21.4 (147.0)	0.93 [29.0]
*tt* **-3b**	148.9	2.71 [0.7]	148.2	2.62 [0.8]	147.9	2.61 [0.8]	147.8	2.66 [0.8]
*cc* **-4b**	−20.6	0.00 [89.2]	−21.0	0.00 [87.2]	−21.3	0.00 [85.7]	−21.4	0.00 [84.7]
*ct* **-4b**	−16.2 (141.2)	1.67 [10.7]	−17.3 (141.6)	1.55 [12.7]	−18.2 (142.2)	1.48 [14.1]	−18.8 (142.5)	1.43 [15.1]
*tt* **-4b**	146.9	4.24 [0.1]	146.1	3.87 [0.1]	145.9	3.62 [0.2]	145.8	3.45 [0.3]
*cc* **-5b**	−20.3	0.00 [92.9]	−21.0	0.00 [91.5]	−21.8	0.00 [88.8]	−22.1	0.00 [86.3]
*ct* **-5b**	−15.0 (139.2)	1.93 [7.1]	−16.1 (139.5)	1.82 [8.4]	−17.1 (140.1)	1.65 [11.0]	−17.8 (140.6)	1.51 [13.5]
*tt* **-5b**	146.6	4.64 [0.0]	146.4	4.18 [0.1]	146.4	3.79 [0.1]	146.5	3.49 [0.2]

^a^The percentage abundances of individual conformers in the equilibrium mixture of three conformers of each compound are shown in square brackets

The replacement of Y = O by a heavier group 16 heteroatom leads to a gradual increase in the twist angle of heteroaryl substituents relative to the C=X group for the *tt*-conformers in vacuum. Their *τ* values become smaller with the growing atomic radius of Y, which means that the substituents are more and more distorted from coplanarity with the C=X group. The same trend in *τ* is observed for the trans-like heteroaryl ring of *ct*-conformers. The cis-like heteroaryl ring displays less regular changes in *τ* but the total inclination between the planes of both heteroaryl rings still grows while Y descends through group 16. For the *cc*-conformers, the replacement of oxygen by a heavier Y heteroatom results in irregular changes of *τ*. In this case, keeping balance between some additional subtle effects, such as intramolecular X⋯Y and H⋯H interactions [28], may be decisive.

The presence of a solvent essentially strengthens the effect of Y on *τ*, if compared to the corresponding *τ* values detected for the *ct*- and *tt*-conformers in vacuum. The resulting solvent effect is, however, rather minor: the inclination between the planes of heteroaryl rings in the solvated *ct*- and *tt*-conformers grows by merely several degrees. The solvent effect does not hold for *ct*-**1a** and *tt*-**1a**, which retain their planar geometry in solvents. The inclination between the planes of heteroaryl rings is usually augmented by the increase of solvent polarity. **1a**, **1b**, **2a**, and **2b** in the *cc*-conformation show, however, the reverse relationship between the inclination of heteroaryl rings and the solvent dielectric constant *ε*.

The relative stability of individual conformers of the investigated compounds was established using the differences Δ*G* in the Gibbs free energies of the conformers at 298.15 K. For each compound, its most stable conformer is characterized by Δ*G* = 0, whereas those possessing higher (i.e., less negative) Gibbs free energies than the preferred conformer demonstrate Δ*G* > 0. An inspection of the Δ*G* values presented for the isolated molecules in Tables 1 and 2 reveals that the *ct*-conformation is the most energetically favorable if Y = O. Unlike **1a** and **1b**, all the remaining compounds prefer the *cc*-conformation, while their *tt*-conformers are least stable. The growing atomic radius of Y along the series **2a**–**5a** and **2b**–**5b** results in increases of Δ*G* for the *ct*- and *tt*-conformers. For that reason, the compounds with Y = Te, Po exhibit negligible percentage abundances of their *tt*-conformers relative to the *cc*-conformers (the conformer abundances are based on the Boltzmann distribution of conformers in their equilibrium mixture at 298.15 K; see section S1). The preferred conformers of the investigated compounds usually make dominant contributions to the corresponding equilibrium mixtures of three conformers. The lowest percentage abundances of the preferred conformer are found for **2a** and **2b** (38.8% and 48.8%, respectively), which results from the small Δ*G* values of their *ct*-conformers.

The presence of a solvent essentially does not change the sequences of conformers ordered relative to their stability. Such a solvent effect occurs for all the investigated compounds with one exception. For **1b** solvated in dichloromethane, the sequence of higher-energy conformers is swapped, compared to the ordering of such conformers in vacuum and in two solvents of lower polarity. The solvent effect on the percentage abundances of preferred conformers is non-uniform, with both increases and decreases in abundance observed while the solvent polarity is growing. For instance, the percentage abundance of *ct*-**1a** is enhanced in polar solvents, whereas the opposite situation is observed for *ct*-**1b**. Nevertheless, a general finding can be made that an increase in the solvent polarity leads to an additional stabilization of *cc*-conformation for **1a**–**3a** and **1b**–**3b**. In consequence, the percentage abundances of these *cc*-conformers go up while the solvent varies from benzene to chloroform and then to dichloromethane. The solvents produce the reverse effect on the *cc*-conformation of **4a**, **4b**, **5a**, and **5b**. For these compounds, their *ct*-conformers experience an effective stabilization by the solvents with higher *ε*. This is accompanied by the corresponding increases in the percentage abundances of *ct*-conformers at the expense of *cc*-conformer abundances.

### Frontier molecular orbitals

Among frontier molecular orbitals, the highest occupied molecular orbital (HOMO) and the lowest unoccupied molecular orbital (LUMO) are particularly important in describing intrinsic molecular properties. Therefore, it is interesting to inspect the heteroatom and solvent effects on the HOMO and LUMO of **1a**–**5a** and **1b**–**5b**.

**Fig. 1:**
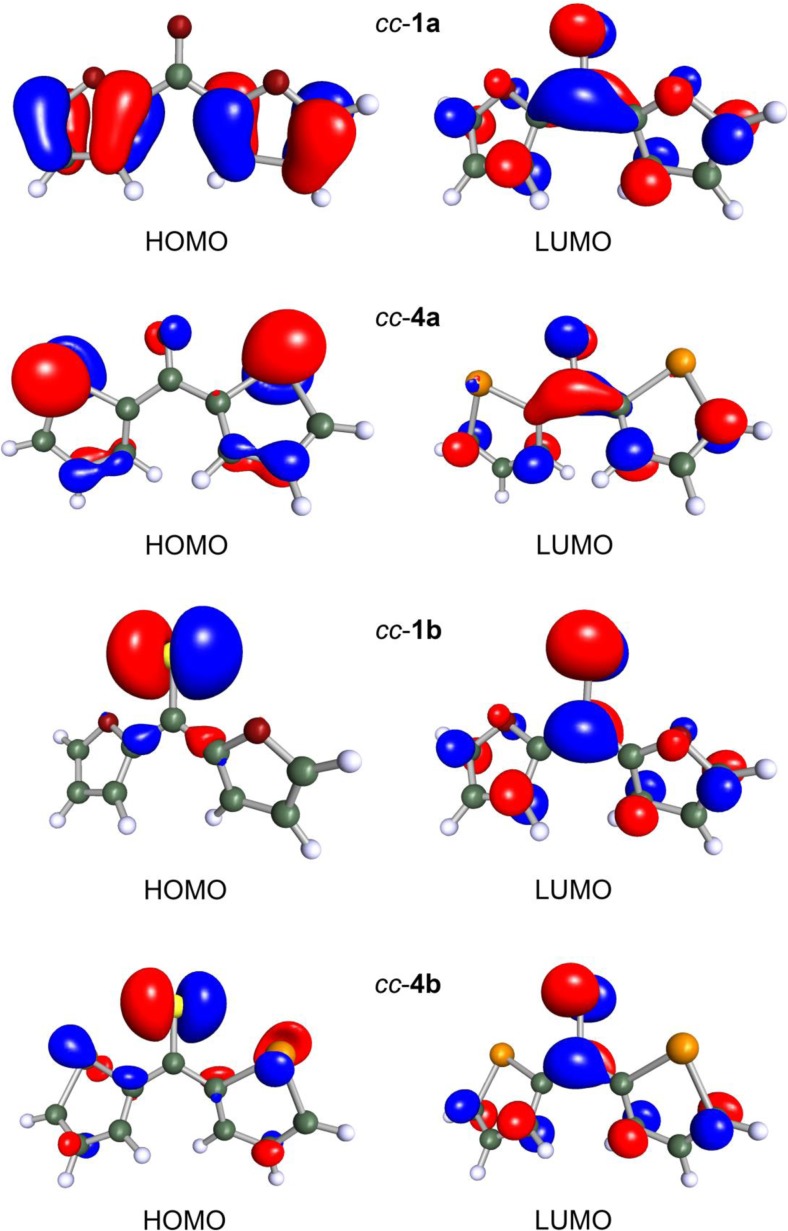
Frontier molecular orbitals of **1a**, **1b**, **4a**, and **4b** adopting the *cc*-conformation. Contours of these orbitals are plotted with an isovalue of 0.05 a.u.

Figure 1 presents the plots of HOMO and LUMO contours for a part of the investigated molecules. The molecules shown in this figure are selected in a way that allows us to detect trends in the heteroatom effect. For **1a**–**3a**, their HOMO shows a purely *π* character and it is delocalized over the heteroaryl substituents. The HOMO of the ketones with heavier ring heteroatoms (Y = Te, Po) possesses substantial contributions from the *σ*-based orbitals belonging to these heteroatoms, and a minor share of X-atom lone pair orbital. The LUMOs of all ketones demonstrate common features. The contribution of the π* orbital of carbonyl group predominates and the LUMOs also spread over the π*-type orbitals of heteroaryl substituents. In the case of the thioketones, their HOMO is centered mainly on the S atom of the thiocarbonyl group. In addition to the lone pair orbital of this S atom, the heavier ring heteroatoms (Y = Te, Po) also provide notable contributions to the HOMO. The LUMO of **1b**–**5b** is fairly similar to that of the ketones.

**Fig. 2:**
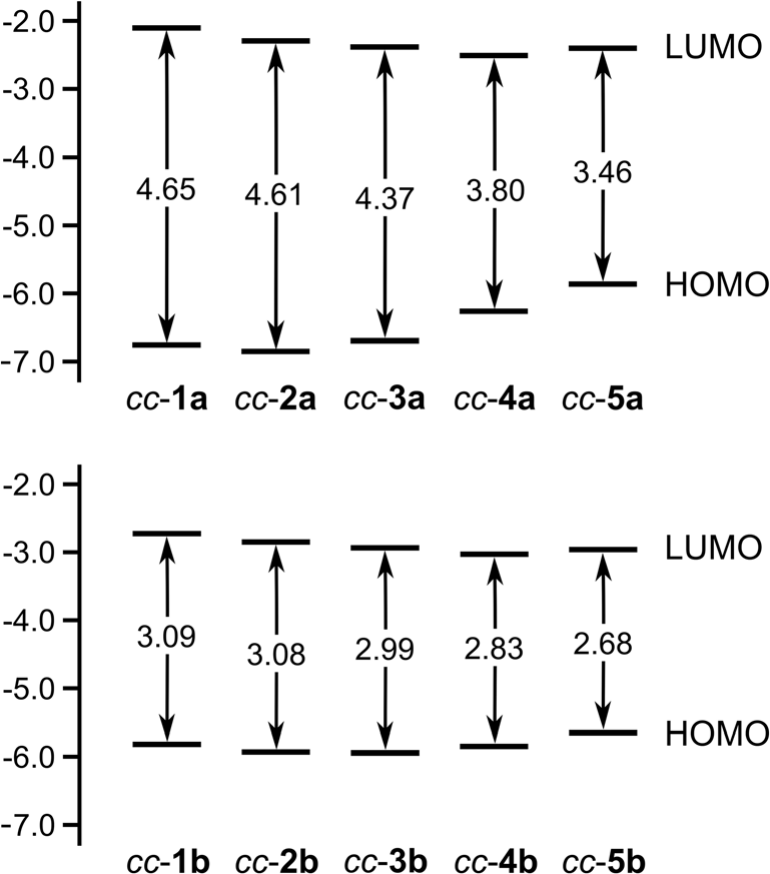
Diagrams illustrating the highest occupied molecular orbital (HOMO) and the lowest unoccupied molecular orbital (LUMO) levels of the *cc*-conformers of **1a**–**5a** and **1b**–**5b** in vacuum. The energy gaps between these levels are also shown. All energies are given in eV

Figure 2 presents the HOMO and LUMO levels of the isolated *cc*-conformers. The difference between the HOMO and LUMO levels, i.e., the HOMO–LUMO energy gap, is also shown for each *cc*-conformer. It is apparent that the growing size of Y progressively narrows the HOMO–LUMO energy gap of both the ketones and thioketones adopting the *cc*-conformation. Such a finding is essentially valid for the other conformations of the investigated molecules (see Figs. S1 and S2). The impact of the kind of Y on the HOMO–LUMO energy gap of the *cc*-conformers of **1a**–**5a** and **1b**–**5b** mimics the corresponding heteroatom effect observed for a series of chalcophenes C_4_H_4_Y (Y = O, S, Se, Te) [50]. The narrowing of the HOMO–LUMO energy gap of **1a**–**5a** and **1b**–**5b** is the result of shifts in the HOMO and LUMO levels while replacing Y with the consecutive elements of group 16. According to the diagrams in Fig. 2, the HOMO level of the *cc*-conformers with heavy Y heteroatoms tends to lie higher in energy than the HOMO levels of the *cc*-conformers containing light Y. The LUMO becomes in turn more and more stabilized, while moving down group 16 from Y = O to Y = Te. These shifts in the HOMO and LUMO levels lead to a regular decrease in the values of the HOMO–LUMO energy gap as the Y heteroatom gets heavier. However, the planar geometry of *ct*-**1a**, *tt*-**1a**, and the nearly planar geometry of *ct*-**1b** additionally stabilize the LUMO level of these conformers, yielding their HOMO–LUMO energy gaps smaller than those of *ct*-**2a**, *tt*-**2a**, and *ct*-**2b** (see Figs. S1 and S2).

**Fig. 3:**
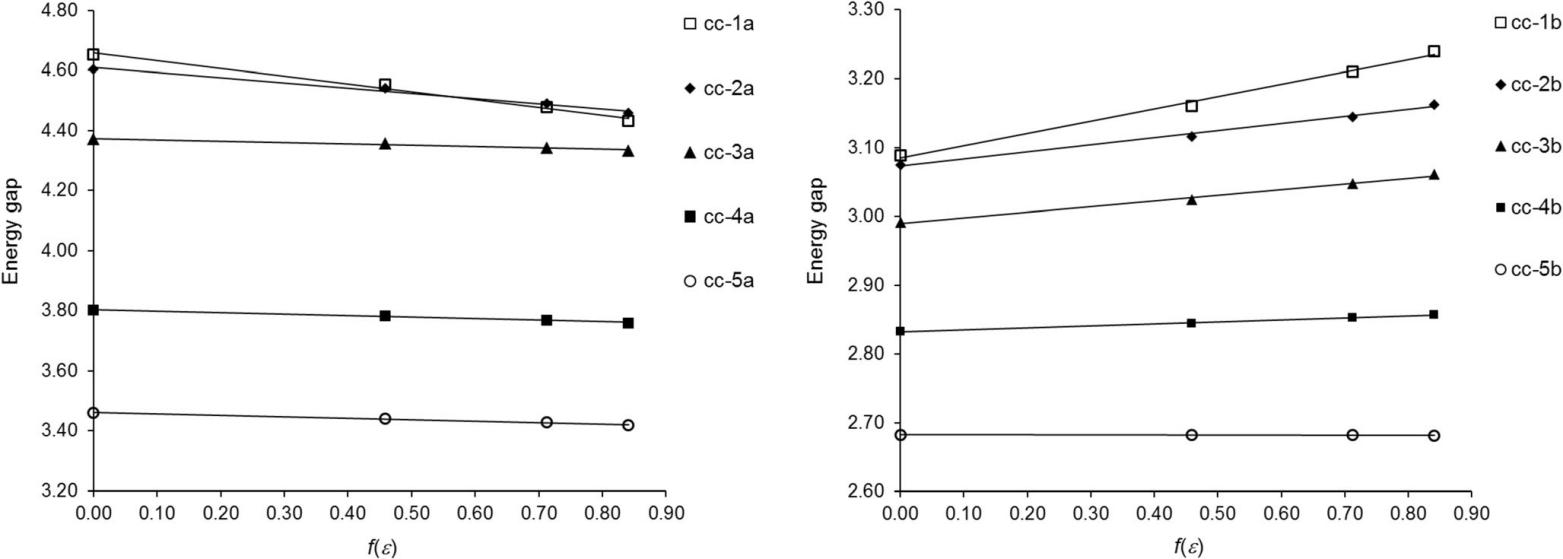
Plots of the HOMO–LUMO energy gap (in eV) against the COSMO correction factor *f*(*ε*) for the *cc*-conformers of **1a**–**5a** and **1b**–**5b** in vacuum and three solvents. The *f*(*ε*) values of vacuum, benzene, chloroform, and dichloromethane amount to 0.00, 0.46, 0.71, and 0.84, respectively

Changes in the HOMO–LUMO gap upon solvation are depicted in Fig. 3. In this figure, the polarity of the solvents is represented by the COSMO *ε*-dependent correction factor *f*(*ε*) = (*ε* – 1)/(*ε* + 0.5) for dielectric screening energy [34]. It is evident from the figure that the changes in the HOMO–LUMO energy gap of the *cc*-conformers are correlated with *f*(*ε*); linear relationships between the HOMO–LUMO energy gap and *f*(*ε*) are detected for the *cc*-conformers, but these relationships differ in the slope of regression line. For each *cc*-conformer, the relationship is established using four points corresponding to the HOMO–LUMO energy gaps of this *cc*-conformer in vacuum and three solvents.

A non-uniform behavior of **1a**–**5a** versus **1b**–**5b** can be seen while analyzing the solvent effect in Fig. 3. For the *cc*-conformers of the ketones, the presence of the solvents provokes a reduction in their HOMO–LUMO energy gap. Moreover, the HOMO–LUMO energy gap narrows gradually upon increasing the polarity of the solvents. This narrowing is predominantly caused by the stabilization of LUMO levels in the solvated *cc*-conformers (see Fig. S3). The HOMO levels are only marginally affected by solvation. The solvents usually have a slight destabilizing influence on these levels. Unlike the ketones, their thiocarbonyl analogs exhibit a broadening in their HOMO–LUMO energy gap upon solvation. The values of the HOMO–LUMO energy gap become larger when the solvent polarity increases. An exception to such a solvent effect occurs for **5b**, whose energy gap remains practically constant in vacuum and three solvents. The broadening of the HOMO–LUMO energy gap of **1b**–**4b** is mainly associated with their HOMO levels, which are shifted to lower (that is, more negative) energies upon solvation (see Fig. S4). The HOMO contours presented in Fig. 1 can serve as a rough rationalization for this shift. The HOMO of *cc*-**1b** and *cc*-**4b** resides mainly on the thiocarbonyl S atom, and the resulting polarization of the HOMO toward the S atom suggests a greater stabilization by more polar solvents. In the case of the LUMO levels of **1b**–**5b**, their stabilization is triggered by the solvents. Unsurprising, this effect is common to both the ketones and thioketones, which is due to the striking similarity of their LUMOs. The aforementioned trends in the solvent effects on the HOMO–LUMO energy gap of the *cc*-conformers of **1a**–**5a** and **1b**–**5b** are also valid for the *ct*- and *tt*-conformers of these compounds.

### Dipole moment

The molecular dipole moment, *μ*, is an essential quantity describing the spatial distribution of electron charge within a molecule. It is known that the magnitude and direction of *μ* is sensitive to molecular size and shape, and, in consequence, different conformers of a molecule can have very different dipole moments. This happens to the molecules of **1a**–**5a** and **1b**–**5b**, which can adopt three conformations, each exhibiting its own abundance at 298.15 K. Therefore, it is reasonable to characterize the dipole moment of every single compound of interest by using its conformationally weighted dipole moment *μ*_cw_ (a methodology for calculating *μ*_cw_ is proposed in section S1). The calculated values of *μ*_cw_ for **1a**–**5a** and **1b**–**5b** are shown in Table 3.

**Table 3:** Conformationally weighted dipole moments (*μ*_cw_ in Debye), polarizabilities (*α*_cw_ in Å^3^) and polarizability anisotropies (Δ*α*_cw_ in Å^3^) for **1a**–**5a** and **1b**–**5b** in vacuum and solvents

Compound	Medium											
	Vacuum			Benzene			Chloroform			Dichloromethane		
	*μ*_cw_	*α*_cw_	Δ*α*_cw_	*μ*_cw_	*α*_cw_	Δ*α*_cw_	*μ*_cw_	*α*_cw_	Δ*α*_cw_	*μ*_cw_	*α*_cw_	Δ*α*_cw_
**1a**	3.72	18.2	14.5	3.81	21.2	16.7	3.84	23.5	18.4	3.87	24.9	19.5
**2a**	3.60	22.4	15.8	3.64	26.5	17.9	3.68	29.5	19.5	3.71	31.4	20.5
**3a**	3.50	24.7	16.8	3.51	29.3	19.0	3.51	32.9	20.6	3.52	35.0	21.6
**4a**	2.78	29.1	18.9	2.79	34.8	21.3	2.79	39.2	23.0	2.79	42.0	24.0
**5a**	1.98	31.5	20.0	1.99	38.2	22.4	2.01	43.4	24.0	2.02	46.7	25.0
**1b**	4.03	22.7	17.2	4.04	27.3	20.7	4.08	30.9	23.6	4.11	33.2	25.5
**2b**	3.86	26.7	17.9	3.88	32.2	21.4	3.90	36.5	24.2	3.91	39.3	26.1
**3b**	3.69	29.0	19.4	3.69	35.2	23.1	3.69	40.1	26.1	3.70	43.2	28.1
**4b**	3.02	33.4	22.9	3.03	40.8	27.0	3.03	46.7	30.2	3.03	50.5	32.3
**5b**	2.34	35.9	24.4	2.35	44.3	28.7	2.37	51.1	32.0	2.39	55.5	34.1

We begin by discussing the calculated *μ*_cw_ values for the molecules of **1a**–**5a** and **1b**–**5b** in vacuum. The *μ*_cw_ values of the ketones range between 1.98 D and 3.72 D. The effect of the type of ring heteroatom on these values is clearly evident; there is a gradual decrease in *μ*_cw_ while Y descends through group 16. The same effect occurs for the thioketones. Their range of *μ*_cw_ is shifted toward higher values, but it is still of similar width to that found for the ketones. The effect of Y on *μ*_cw_ can be rationalized in terms of variations in the electronegativity of Y, and in the resultant local dipole moment of a heteroaryl substituent. The decrease of Y electronegativity along the series from Y = O to Y = Te is associated with a decrease in the local dipole moment of the corresponding heteroaryl substituent. The local dipole moment of a heteroaryl ring with Y = O–Se acts from the Y heteroatom toward the ring center (assuming that the dipole vector is directed toward the center of positive partial charge). Two such oriented dipole moments added to the local dipole vector of the C=X group generate a large molecular dipole moment for the *cc*-conformers of **1a**–**3a** and **1b**–**3b**. Our calculations of *μ* for tellurophene predict a negligible value of 0.04 D, while polonophene possesses larger *μ* (0.44 D) yet its orientation is reversed. The local dipole moments of the polonophen-2-yl rings compensate to a certain degree the local dipole moment of the C=X group for the *cc*-conformers of **5a** and **5b**. The high abundance of these conformers has, in turn, a direct effect on *μ*_cw_, and, therefore, the values of *μ*_cw_ for **5a** and **5b** are smallest in both series of compounds.

The comparison of *μ*_cw_ for every **1a**–**5a** in vacuum with *μ*_cw_ for the corresponding thiocarbonyl analog reveals that the former is generally smaller in magnitude than the latter. This indicates that the distribution of electron charge is less polarized within the molecules of the ketones than within the molecules of the corresponding thioketones. The *μ*_cw_ values of **1a**–**5a** turn out to be smaller despite the fact that the electronegativity of oxygen is larger than that of sulfur and, in principle, the C=O group as such is more polarized than the C=S group [51]. In this case, the electron donor–acceptor properties of heteroaryl rings become a factor reinforcing the charge separation within the molecules of **1b**–**5b**. An increase in the magnitude of molecular dipole moment upon replacing C=O by C=S was previously observed for diaryl ketones [52] and for uracil and its thio-analogs [53].

Having analyzed the *μ*_cw_ values in vacuum, we turn our attention to the solvent effect on *μ*_cw_. It is evident from Table 3 that the *μ*_cw_ values of the investigated compounds in three solvents are always larger than those characterizing these compounds in vacuum. This indicates a stronger charge separation within the solvated molecules of **1a**–**5a** and **1b**–**5b** than within the corresponding isolated molecules. This is because the charge distribution of each solute molecule polarizes the solvent, which, in turn, acts back on the solute molecule, enhancing its intramolecular charge separation and magnitude of molecular dipole moment. As states above, *μ*_cw_ grows if the phase of each compound has been altered from vacuum to solution, and additionally, the resulting growth of *μ*_cw_ is enhanced by the increasing polarity of the solvents. Thus, the largest *μ*_cw_ values are observed for the investigated compounds solvated by dichloromethane. The growth of *μ*_cw_ is, however, rather minor and does not exceed several percent, compared to *μ*_cw_ in vacuum (the maximal *μ*_cw_ growth amounting to 4.1% is observed for **1a** in dichloromethane). This also suggests that there will be a modest increase in the intermolecular solute–solvent interaction if the solvent is changed from benzene to dichloromethane.

There are available experimental values of molecular dipole moment for three out of ten investigated compounds. Experimental measurements were made for **1a** (3.65 D), **2a** (3.71 D), and **2b** (3.75 D), all in benzene solution [54]. The *μ*_cw_ values calculated for **1a**, **1b**, and **2b** in benzene reproduce the corresponding experimental dipole moments with an accuracy of 0.16 D or better. The agreement of the trend in the *μ*_cw_ values with the experimental results was, however, successful only in part. The *μ*_cw_ values of **2a** and **2b** mimic the experimentally measured growth of molecular dipole moment. The *μ*_cw_ values of **1a** and **2a**, by contrast, do not follow the experimental trend. One should bear in mind that both the experimental dipole moments and *μ*_cw_ characterize the equilibrium mixtures of conformers, so the aforementioned disagreement may result even from a minor difference in the abundance of individual conformers.

Next, the analysis of the molecular dipole moments of **1a**–**5a** and **1b**–**5b** will be deepened by inspecting the *μ* values of individual conformers. The *μ* values of all investigated conformers are collected in Table 4. It is apparent that there are noticeable differences in *μ* between individual conformers of every compound. For **1a**–**3a** and their thiocarbonyl counterparts, the *cc*-conformation exhibits the largest values of *μ*, whereas the *tt*-conformers possess the smallest *μ* values. The molecular dipole moments of **4a**, **4b**, **5a**, and **5b** are also sensitive to conformational changes, but these compounds show an increase in their *μ* values while passing from the *cc*-conformation to the *ct*-conformation and further to the *tt*-conformation. For all conformers, the spatial orientation of their *μ* vectors is determined, to a great extent, by the orientation of the local dipole moment of their C=O or C=S group (this local dipole moment is directed from the O or S atom toward the C atom). The relation between the increment of *μ* and the kind of conformation can be understood in terms of the local dipole moments ascribed to the heteroaryl fragments and the C=O and C=S groups. The orientations of these local dipole moments for the *cc*-conformers of **1a**–**5a** and **1b**–**5b** have been discussed earlier in this subsection. The *tt*-conformers of **1a**–**3a** and **1b**–**3b** possess the smallest *μ* values because the local dipole moments of their heteroaryl fragments and of their C=O and C=S groups are directed oppositely. These local dipole moments demonstrate an approximately concordant direction for the *tt*-conformers of **5a** and **5b**, and, therefore, their *tt*-conformation exhibits the largest *μ* values.

**Table 4:** Dipole moments (*μ* in Debye) for the conformers of **1a**–**5a** and **1b**–**5b** in vacuum and solvents

Conformer	*μ* ^a^	Conformer	*μ* ^a^
*cc* **-1a**	4.75/5.67/6.38/6.82	*cc* **-1b**	4.72/5.86/6.79/7.39
*ct* **-1a**	3.91/4.69/5.27/5.64	*ct* **-1b**	4.10/5.10/5.92/6.44
*tt* **-1a**	2.90/3.45/3.87/4.12	*tt* **-1b**	3.35/4.14/4.77/5.19
*cc* **-2a**	4.03/4.89/5.55/5.97	*cc* **-2b**	4.14/5.17/6.02/6.57
*ct* **-2a**	3.36/4.09/4.66/5.01	*ct* **-2b**	3.62/4.52/5.26/5.74
*tt* **-2a**	2.69/3.27/3.72/4.00	*tt* **-2b**	3.09/3.84/4.45/4.85
*cc* **-3a**	3.65/4.47/5.11/5.52	*cc* **-3b**	3.81/4.79/5.60/6.13
*ct* **-3a**	3.20/3.93/4.50/4.87	*ct* **-3b**	3.46/4.36/5.10/5.59
*tt* **-3a**	2.80/3.44/3.94/4.26	*tt* **-3b**	3.17/3.98/4.64/5.08
*cc* **-4a**	2.76/3.42/3.96/4.30	*cc* **-4b**	3.00/3.83/4.52/4.98
*ct* **-4a**	2.94/3.67/4.26/4.63	*ct* **-4b**	3.19/4.08/4.83/5.33
*tt* **-4a**	3.24/4.02/4.63/5.03	*tt* **-4b**	3.60/4.58/5.40/5.94
*cc* **-5a**	1.93/2.32/2.66/2.89	*cc* **-5b**	2.28/2.87/3.39/3.74
*ct* **-5a**	2.85/3.62/4.26/4.68	*ct* **-5b**	3.10/4.06/4.90/5.47
*tt* **-5a**	3.82/4.89/5.77/6.35	*tt* **-5b**	4.17/5.50/6.64/7.43

^a^The *μ* values are listed in the following media: vacuum/benzene/chloroform/dichloromethane

The effect on *μ* exerted by the solvents is not of identical magnitude for all conformers. For each compound, the difference between the *μ* values of its most polar and least polar conformers increases upon solvation, and such a difference keeps growing with increasing solvent polarity. The conformers of **1b**–**5b** exhibit higher growth of *μ* upon solvation than the corresponding ketone conformers. This is in agreement with previous findings reported for formaldehyde and thioformaldehyde solvated in water [55]. The polarity of the C–S π bond in thioformaldehyde is increased to a greater extent than that observed for the C–O π bond in formaldehyde.

Finally, it is instructive to relate the abundances of solvated conformers in their equilibrium mixtures to the changes of their *μ* values upon solvation. In principle, it can be expected that a conformer possessing a high *μ* value becomes more favorable in solvents of high polarity [56]. Such a relationship exists for the solvated conformers of **1a**–**5a** and **1b**–**5b**, and their percentage abundances shown in Tables 1 and 2 provide evidence confirming this. Among the three conformations of **1a**–**3a** and **1b**–**3b** in vacuum, the *cc*-conformers exhibit the highest *μ* values; hence, these conformers become more and more abundant in the solvents of increasing polarity. The same concerns the *tt*-conformers of **4a**, **4b**, **5a**, and **5b**, although these conformers are still much higher in energy than the preferred conformers, and, therefore, the abundances of the *tt*-conformers rise extremely slowly (their tabulated percentage abundances sometimes seem not to grow, but this is because the percentage abundances have been rounded to one digit after the decimal point).

### Polarizability

The molecular average dipole polarizability *α* and the polarizability anisotropy Δ*α* measure the deformation of charge distribution within a molecule under an applied external electric field. Here, the importance of *α* and Δ*α* is attributed to the fact that these quantities may be good indicators of how the deformation of charge distribution within the investigated molecules is affected by the kind of Y and the presence of solvent. Similarly to *μ*_cw_, the *α* and Δ*α* values of three conformers of each compound are subjected to conformational weighting in order to obtain *α*_cw_ and Δ*α*_cw_. The *α*_cw_ and Δ*α*_cw_ values calculated for **1a**–**5a** and **1b**–**5b** are appended to Table 3.

**Fig. 4:**
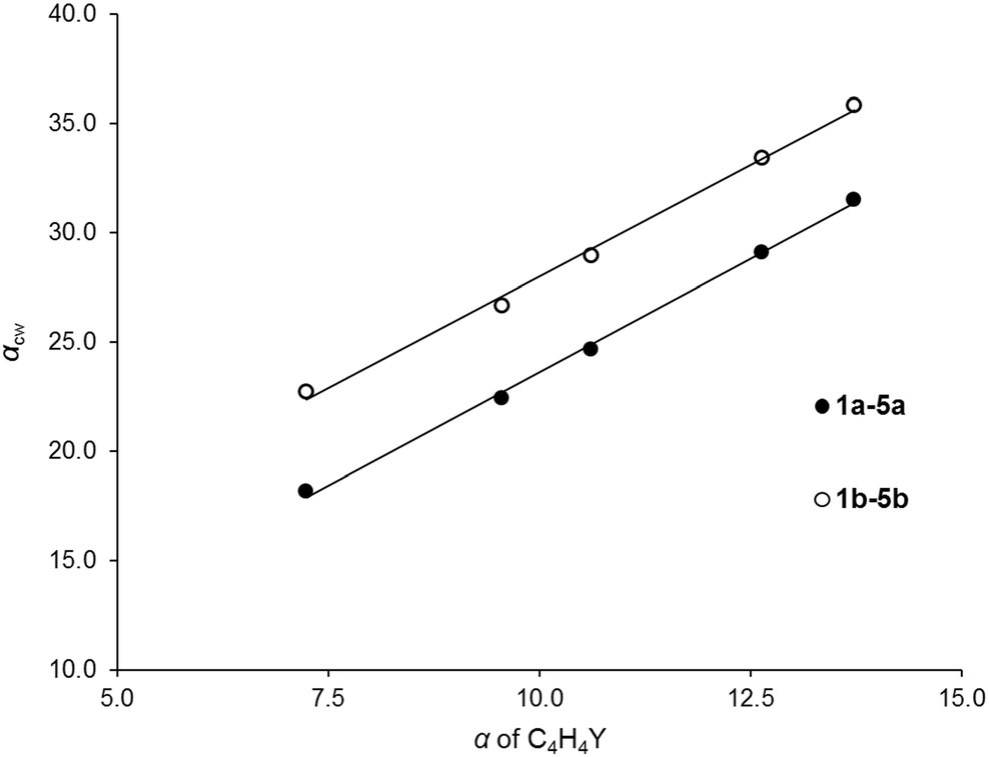
Plot of the conformationally weighted polarizability of **1a**–**5a** and **1b**–**5b** in vacuum (*α*_cw_ in Å^3^) against the polarizability of parent heterocyclic molecules C_4_H_4_Y in vacuum (*α* in Å^3^)

The Y heteroatom effect on *α*_cw_ and Δ*α*_cw_ is the opposite of that detected for *μ*_cw_. For each series of compounds, the replacement of oxygen by a heavier group 16 element at the Y position increases the values of *α*_cw_ and Δ*α*_cw_ gradually. The distribution of electron charge in **5a** and **5b** undergoes the greatest distortion caused by an external electric field. The trend in *α*_cw_ can be interpreted in terms of changes in the molecular polarizabilities of five-membered heterocycles C_4_H_4_Y. Figure 4 proves that the *α*_cw_ values of **1a**–**5a** in vacuum are linearly correlated with the polarizabilities of isolated chalcophenes. The same applies to the thioketones. The *α*_cw_ values of **1b**–**5b** are shifted toward more positive values due to the higher atomic polarizability of the thiocarbonyl S atom compared to the O atom of the C=O group. The molecular polarizabilities of C_4_H_4_Y heterocycles are, in turn, in excellent linear correlation with the atomic polarizabilities of the corresponding Y atoms [57]. This indicates that the evolution of *α*_cw_ along the series of the ketones or thioketones investigated here is determined mainly by the kind of Y heteroatom.

The heteroatom effect on polarizability can also be rationalized in terms of HOMO–LUMO energy gap and molecular size for a fixed type of molecular conformation. In principle, the polarizability of a molecule is expected to be enhanced with the decreasing HOMO–LUMO energy gap and growing size of the molecule [58]. As noted earlier in this work, replacing Y with a heavier and heavier group 16 element leads to reduction of the HOMO–LUMO energy gap. This reduction is indeed inversely related with *α* for the isolated *cc*-conformers of **1a**–**5a** and **1b**–**5b** (see Fig. S5). Additionally, there is an approximately linear relationship between *α* and the molecular volume of the isolated *cc*-conformers of the investigated compounds (Fig. S5). This reflects the well-known proportionality between molecular polarizability and molecular size [58]. The use of the molecular volume of the *cc*-conformers to estimate their molecular size turns out to be successful for providing a clear correlation with *α*. On the one hand, this may be a consequence of the structural similarity of the *cc*-conformers. On the other hand, the existence of a general relationship between polarizability and volume was previously postulated, although such a relationship did not fulfill an ideal linear correlation [59].

The *α*_cw_ and Δ*α*_cw_ values of **1a**–**5a** and **1b**–**5b** in three solvents are reported in Table 3. An inspection of these values reveals that they are affected by the presence and kind of solvents. Compared to *α*_cw_ and Δ*α*_cw_ of **1a**–**5a** and **1b**–**5b** in vacuum, the *α*_cw_ and Δ*α*_cw_ values of solvated molecules are noticeably larger, and they increase further with the growing polarity of solvents. The growth of *α*_cw_ and Δ*α*_cw_ for the molecules solvated in dichloromethane can reach even 50% of the *α*_cw_ and Δ*α*_cw_ values for the corresponding isolated molecules. The growing solvent polarity facilitates the charge separation within the molecules of **1a**–**5a** and **1b**–**5b**, which, in turn, enhances the deformation of their charge distribution under an external electric field. For **1a**–**5a**, the solvent effect may also be explained by the continuous decrease of the HOMO–LUMO energy gap (see Fig. 2). Such an explanation does not apply to the thioketones, for which the growth of their molecular volume upon solvation determines the increase of *α* (for details, consult Figs. S6 and S7).

The molecular polarizability is only very slightly sensitive to the conformation adopted by the molecules of **1a**–**5a** and **1b**–**5b**. Therefore, the *α* values of three conformers of each compound show much lower variability than the *μ* values. From an experimental perspective, the *α* values seem of little use to unambiguously distinguish the conformers from each other.

## Summary

Density functional theory calculations of geometry, energetics, frontier molecular orbitals, dipole moment and polarizability for formaldehyde and thioformaldehyde symmetrically disubstituted with five-membered heteroaromatic rings containing a single group 16 heteroatom have yielded a detailed picture of variations in these molecular properties as a result of replacing the ring heteroatom (i.e., oxygen) by a heavier element, up to polonium. Furthermore, embedding the molecules of the investigated compounds in a continuum with a small dielectric constant of benzene, chloroform, or dichloromethane has served as a computational tool for establishing to what extent the presence of these low polarity solvents affects the aforementioned properties.

The effect of ring heteroatom on the preferred geometrical orientation of heteroaryl rings is non-uniform for **1a**–**5a** and **1b**–**5b**. The furan-2-yl substituents of **1a** and **1b** favor the *ct*-conformation, whereas the heavier Y heteroatoms tend to prefer the *cc*-conformation. The presence of a solvent further diversifies the conformational behavior of the investigated molecules. The three solvents stabilize the *cc*-conformation of the heteroaryl rings containing Y = O–Se and destabilize this conformation if Y = Te, Po. Such a solvent effect may be rationalized by the analysis of molecular dipole moment for individual conformers. Even if the solvents display low polarity, they have a noticeable influence on the percentage abundances of three conformers in their equilibrium mixture at 298.15 K. The HOMO–LUMO energy gap of **1a**–**5a** and **1b**–**5b** gradually decreases while Y descends through group 16. Two opposite trends in this property are detected for the solvated molecules (**1a**–**5a** versus **1b**–**5b**), which is attributed to different stabilization/destabilization patterns of frontier molecular orbitals in the two series of molecules. The growing size of Y is associated with the decrease of *μ*_cw_ and, simultaneously, with the increases of *α*_cw_ and Δ*α*_cw_. These changes correlate with the electronegativity and polarizability of Y. Both *μ*_cw_ and *α*_cw_ increase when the solvents have been introduced. However, *α*_cw_ is much more sensitive to the growing polarity of the solvents than is *μ*_cw_.

We believe that this work may contribute to a better understanding of the role of heteroatom and solvent in establishing structure–property patterns for organic molecules containing C_4_H_3_Y groups.

## Electronic supplementary material

ESM 1 (DOC 8565 kb)
