# Supplementary material for: Heteroatom and solvent effects on molecular properties of formaldehyde and thioformaldehyde symmetrically disubstituted with heterocyclic groups C4H3Y (where Y = O–Po)
Source: J Mol Model. 2017 Aug 21;23(9):268. doi: 10.1007/s00894-017-3435-4 (PMC5563515; doi:10.1007/s00894-017-3435-4)
Supplement: Supplementary file 1 — (DOC 8565 kb) [file 894_2017_3435_MOESM1_ESM.doc]

**Electronic Supplementary Material**

for

Heteroatom and solvent effects on molecular properties of formaldehyde and thioformaldehyde symmetrically disubstituted with heterocyclic groups C_4_H_3_Y

(where Y = O–Po)

by

Piotr Matczak and Małgorzata Domagała

Department of Theoretical and Structural Chemistry, Faculty of Chemistry, University of Łódź, Pomorska 163/165, 90-236 Lodz, Poland

**S1. Further details of computational methodology**

In the present work, three molecular static electric properties have been computed for the conformers of **1a**–**5a** and **1b**–**5b** according to Eqs. (S1)–(S3). The magnitude of the molecular dipole moment (*μ*) of each conformer has been obtained using the following formula

$\mu=\left(\mu_{x}^{2}+\mu_{y}^{2}+\mu_{z}^{2}\right)^{\frac{1}{2}}$ (S1)

where *μ_i_* (*i = x, y, z*) are the dipole moment components along the *x*, *y*, and *z* axes.

The molecular orientationally-averaged (isotropic) static dipole electronic polarizability (*α*) and polarizability anisotropy (Δ*α*) have been calculated using polarizability tensor elements *α_ij_* (*i, j = x, y, z*) according to the following definitions:

$\alpha=\frac{1}{3}\left(\alpha_{xx}+\alpha_{yy}+\alpha_{zz}\right)$ (S2)

$\Delta\alpha=\frac{1}{\sqrt{2}}\left[\left(\alpha_{xx}-\alpha_{yy}\right)^{2}+\left(\alpha_{yy}-\alpha_{zz}\right)^{2}+\left(\alpha_{zz}-\alpha_{xx}\right)^{2}+6\left(\alpha_{xy}^{2}+\alpha_{xz}^{2}+\alpha_{yz}^{2}\right)\right]^{\frac{1}{2}}$. (S3)

In addition to the calculation of *μ*, *α* and Δ*α* for individual conformers, the values of these properties have been determined for **1a**–**5a** and **1b**–**5b**, taking into account their entire conformational space. For each compound, the *μ*, *α*, and Δ*α* values obtained for its three stable conformations (*cc*, *ct*, *tt*), with one of them doubly degenerated (*ct* and *tc*), are used in the calculations of the resulting conformationally-weighted properties *μ*_cw_, *α*_cw_, and Δ*α*_cw_. For example, the conformationally-weighted molecular dipole moment is expressed as

$\mu_{\text{cw}}=\sum_{i} A_{i}\mu_{i}$ (S4)

where *A_i_* is the fractional abundance of the *i*th conformer and *μ_i_* is the molecular dipole moment of this conformer. The summation *i* runs over three stable conformers (*cc*, *ct*, *tt*).

The fractional abundance of the *i*th conformer is estimated according to the Boltzmann distribution at 298.15K

$A_{i}=\frac{d_{i}\exp\left(\frac{-\Delta G_{i}}{kT}\right)}{\sum_{j} d_{j}\exp\left(\frac{-\Delta G_{j}}{kT}\right)}$ (S5)

where *d_i_* is the degeneracy of the *i*th conformer, Δ*G_i_* is the difference in the Gibbs free energy of the *i*th conformer relative to the Gibbs free energy of the most stable conformer, *k* is the Boltzmann constant, and the temperature *T* is equal to 298.15K. Similarly to *i* in Eq. (S4), the summation *j* in the denominator of Eq. (S5) runs over three stable conformations. The degeneracy of *cc*- and *tt*-conformers amounts to unity, whereas the degeneracy of *ct*-conformers is equal to 2.

The percentage abundance of each conformer (as it is listed in Tables 1 and 2) is obtained from its fractional abundance times 100.

The molecular volume of the *cc*-conformers of **1a**–**5a** and **1b**–**5b** (see Figs. S5– S7) is defined as the volume closed by the van der Waals surface. This surface is in turn delineated by an electron density contour of 0.001 au. The calculations of the molecular volume have been carried out using AIMAll 14.06.21 [1].

**S2. A test of the computational methodology used in the present work**

The accuracy of the computational methodology used in this work to obtain the molecular electric properties of **1a**–**5a** and **1b**–**5b** has been evaluated by performing test calculations for a set of small molecules being the building blocks of **1a**–**5a** and **1b**–**5b**. The molecules of formaldehyde, thioformaldehyde, furan, thiophene, selenophene, and tellurophene are taken into account. For these six molecules there are available experimental values of their molecular dipole moment *μ* and static polarizability *α* (see Table S1). Comparing these experimental values to the corresponding results of B3LYP/def2-QZVPP and B3LYP/def2-QZVPPD calculations allows us to estimate the accuracy of the two computational methods.

The geometries of the six isolated molecules were optimized at the B3LYP/def2-QZVPP level of theory. The molecular dipole moment (*μ*) and static dipole polarizability (*α*) were calculated for the optimized isolated molecules using the B3LYP/def2-QZVPP and B3LYP/def2-QZVPPD methods (for tellurophene the def2-QZVPP and def2-QZVPPD basis sets were replaced by dhf-QZVPP-2c and dhf-QZVPPD-2c, respectively). The calculated values of *μ* and *α* for the test set of molecules are gathered in Table S1. This table also presents a statistical comparison of the calculated results with the reference experimental data. The statistical comparison is based on two metrics of errors in the calculated *μ* and *α* values. The mean signed error (MSE) provides information about systematic errors occurring in the calculated *μ* and *α* values. If the MSE of a given property is positive (negative), then the calculated values of this property are overestimated (underestimated) relative to the reference experimental results. The root mean-squared error (RMSE) is used to characterize the overall performance of each level of theory in predicting *μ* or *α*.

From the MSE(*μ*) and RMSE(*μ*) values in Table S1, it can be deduced that the accuracy of B3LYP combined with both basis sets is very similar. The augmentation of the def2-QZVPP basis set with a small set of diffuse functions leads to only a slight improvement. Based on MSE(*μ*), it is clear that B3LYP tends to underestimate the *μ* values slightly. The RMSE(*μ*) values indicate that B3LYP/def2-QZVPP and B3LYP/def2-QZVPPD perform very well in predicting *μ* for the test set of molecules. The RMSE(*μ*) value of 0.07 D is in fact much smaller than the range of *μ* errors obtained by a variety of DFT methods in a recent benchmark study [2]. The range of *μ* errors reported therein was 0.12–0.13 D.

The augmentation of def2-QZVPP with diffuse functions turns out to be absolutely essential in predicting *α* with satisfactory accuracy. The RMSE(*α*) value for B3LYP/def2-QZVPP is more than twice as large as that for B3LYP/def2-QZVPPD. The latter level of theory yields the *α* values of the six molecules with the RMSE(*α*) value of 0.23 Å^3^. This is better than the range of errors in *α* calculated for a large set of molecules by a variety of DFT methods [2]. B3LYP/def2-QZVPPD tends to underestimate the *α* values of the six molecules slightly (MSE(*α*) < 0).

Based on the results of the test calculations presented above, it can be concluded that B3LYP/def2-QZVPPD predicts the values of *μ* and *α* for the set of six small molecules with great accuracy. Therefore, it is highly likely that this level of theory is able to provide accurate values of *μ* and *α* for the molecules of **1a**–**5a** and **1b**–**5b**.

Table S1. Calculated and experimental dipole moment (*μ* in Debye) and static polarizability (*α* in Å^3^) for six molecules in vacuum. The last two rows give the MSE and RMSE of the calculated *μ* and *α* values from the experimental ones.

| Molecule | B3LYP/def2-QZVPP ^a^ | | B3LYP/def2-QZVPPD ^b^ | | Experiment ^c^ | |
| --- | --- | --- | --- | --- | --- | --- |
|  | *μ* | *α* | *μ* | *α* | *μ* | *α* |
| formaldehyde | 2.36 | 2.51 | 2.38 | 2.69 | 2.33 | 2.77 |
| thioformaldehyde | 1.72 | 4.76 | 1.73 | 5.21 | 1.647 | 5.721 |
| furan | 0.65 | 6.98 | 0.66 | 7.24 | 0.66 | 7.20 |
| thiophene | 0.49 | 9.20 | 0.50 | 9.55 | 0.53 | 9.659 |
| selenophene | 0.37 | 10.13 | 0.38 | 10.60 | 0.39 | 11.08 |
| tellurophene | 0.04 | 12.21 | 0.04 | 12.62 | 0.19 | 12.70 |
| MSE | -0.02 | -0.48 | -0.01 | -0.14 |  |  |
| RMSE | 0.07 | 0.54 | 0.07 | 0.23 |  |  |

^a^ The dhf-QZVPP-2c basis set is used for tellurophene.

^b^ The dhf-QZVPPD-2c basis set is used for tellurophene.

^c^ Experimental values are taken from Refs. [3–6].

**S3. Additional figures**


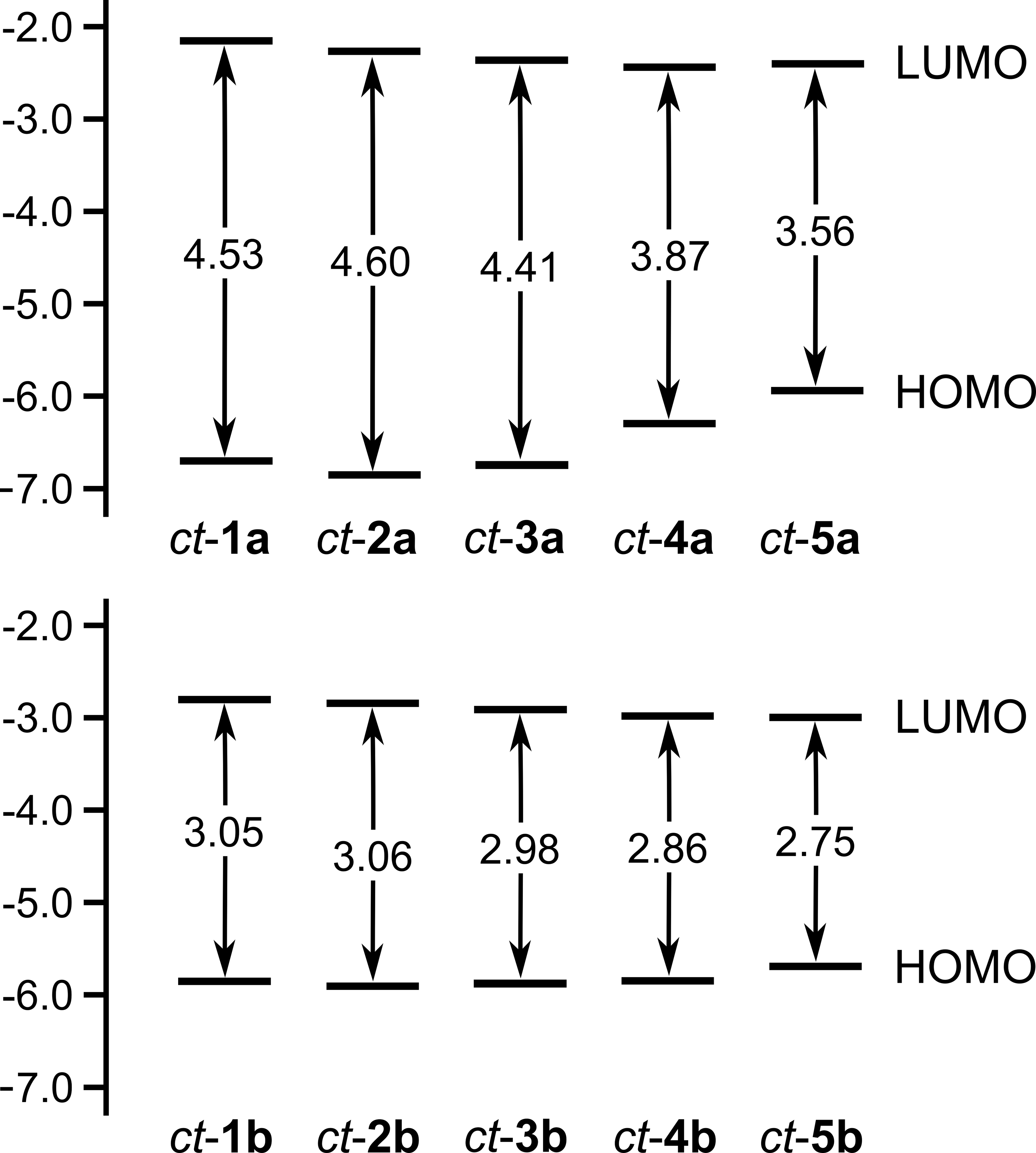


Figure S1. Diagrams illustrating the HOMO and LUMO levels of the *ct*-conformers of **1a**–**5a** and **1b**–**5b** in vacuum. The energy gaps between these levels are also shown. All energies are given in eV.


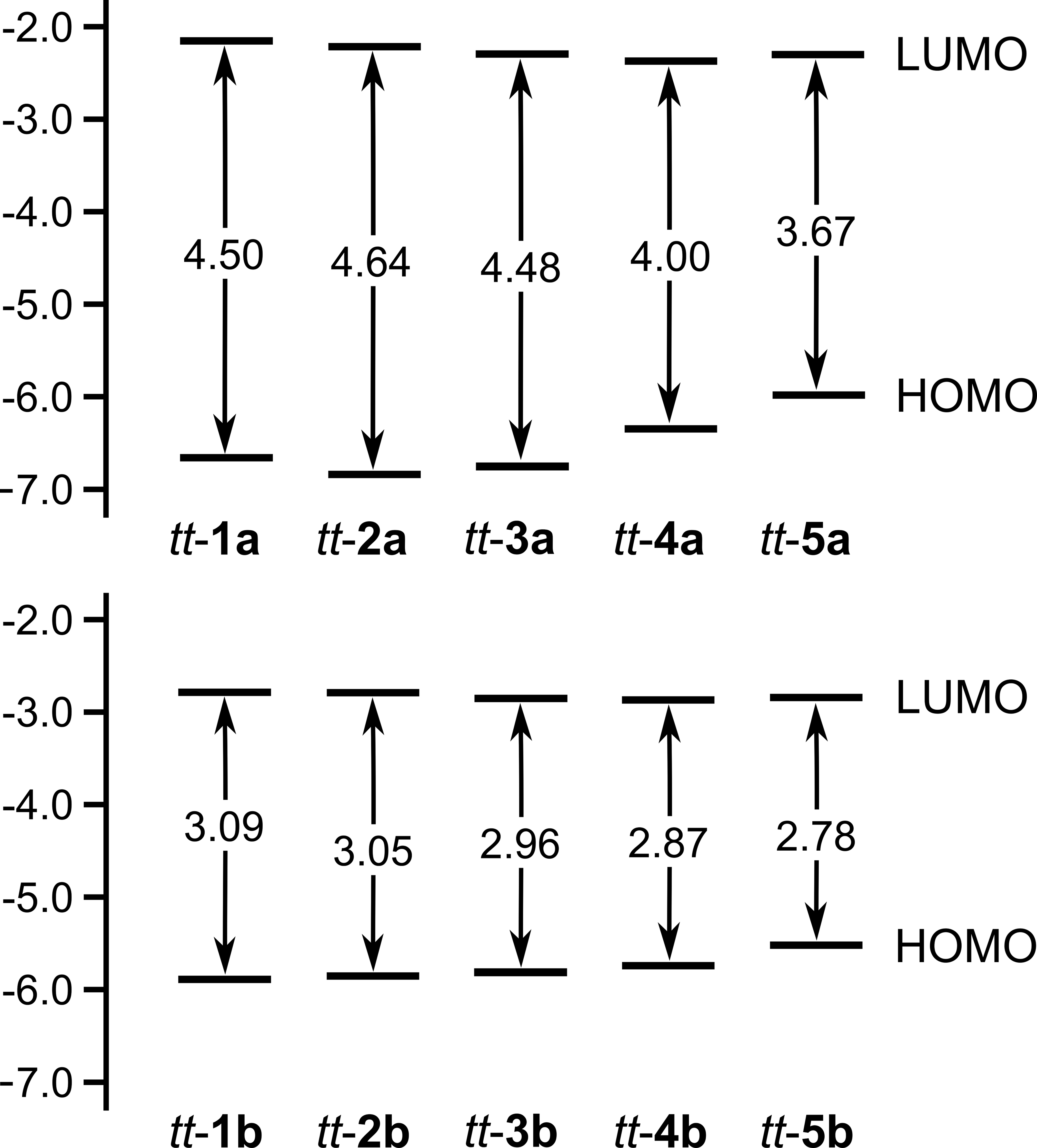


Figure S2. Diagrams illustrating the HOMO and LUMO levels of the *tt*-conformers of **1a**–**5a** and **1b**–**5b** in vacuum. The energy gaps between these levels are also shown. All energies are given in eV.


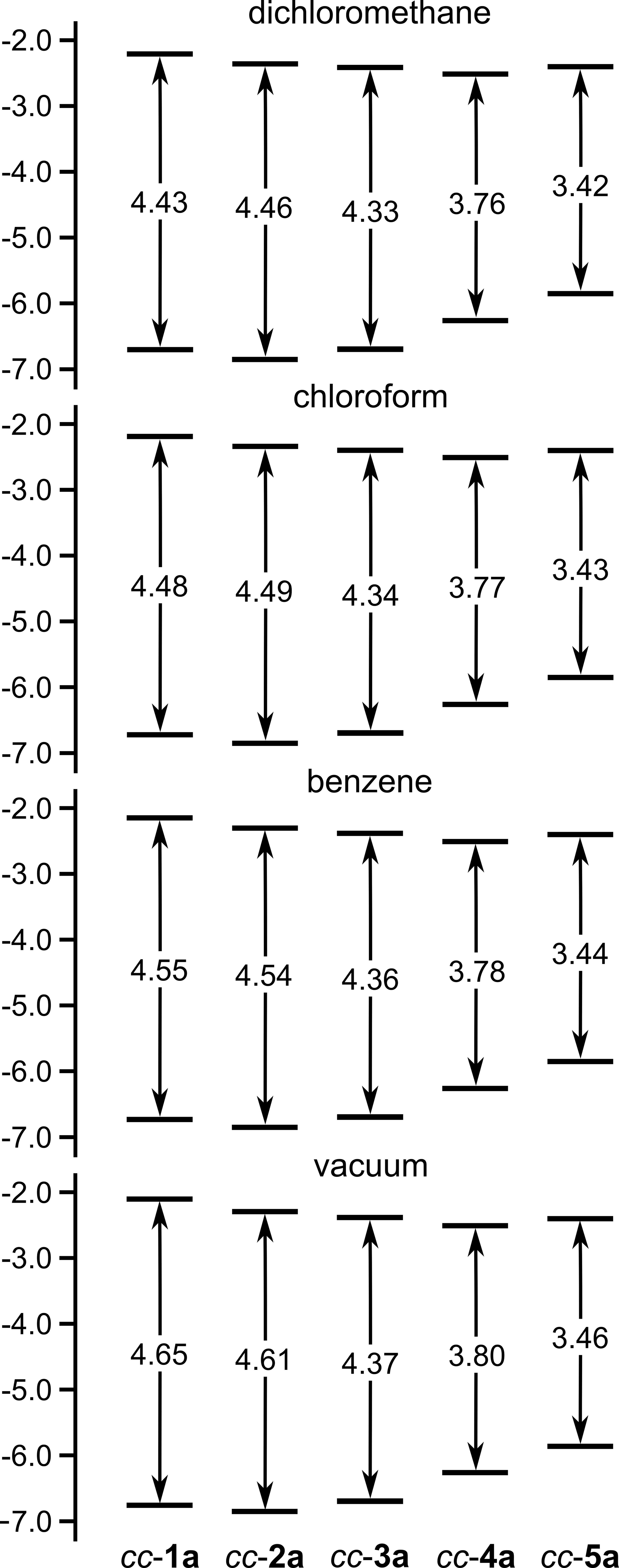


Figure S3. Diagrams illustrating the HOMO and LUMO levels of the *cc*-conformers of **1a**–**5a** in vacuum and three solvents. The energy gaps between these levels are also shown. All energies are given in eV.


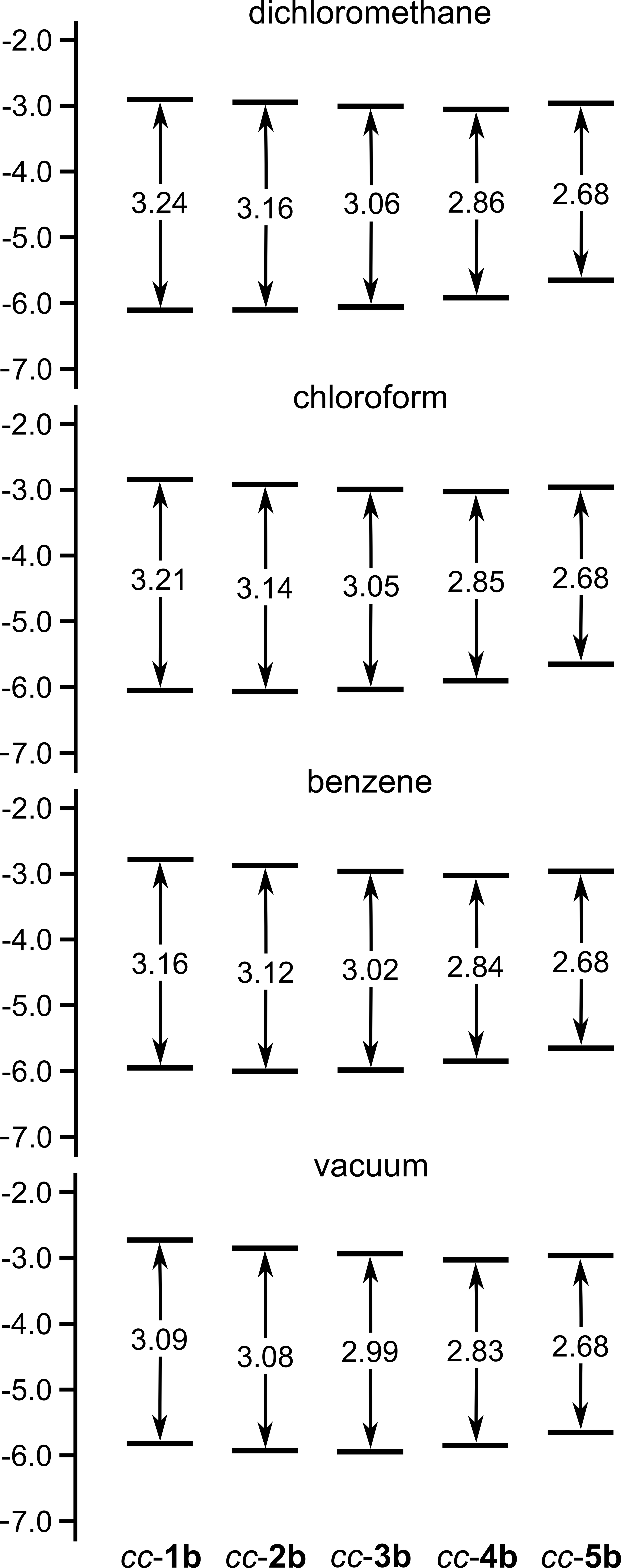


Figure S4. Diagrams illustrating the HOMO and LUMO levels of the *cc*-conformers of **1b**–**5b** in vacuum and three solvents. The energy gaps between these levels are also shown. All energies are given in eV.


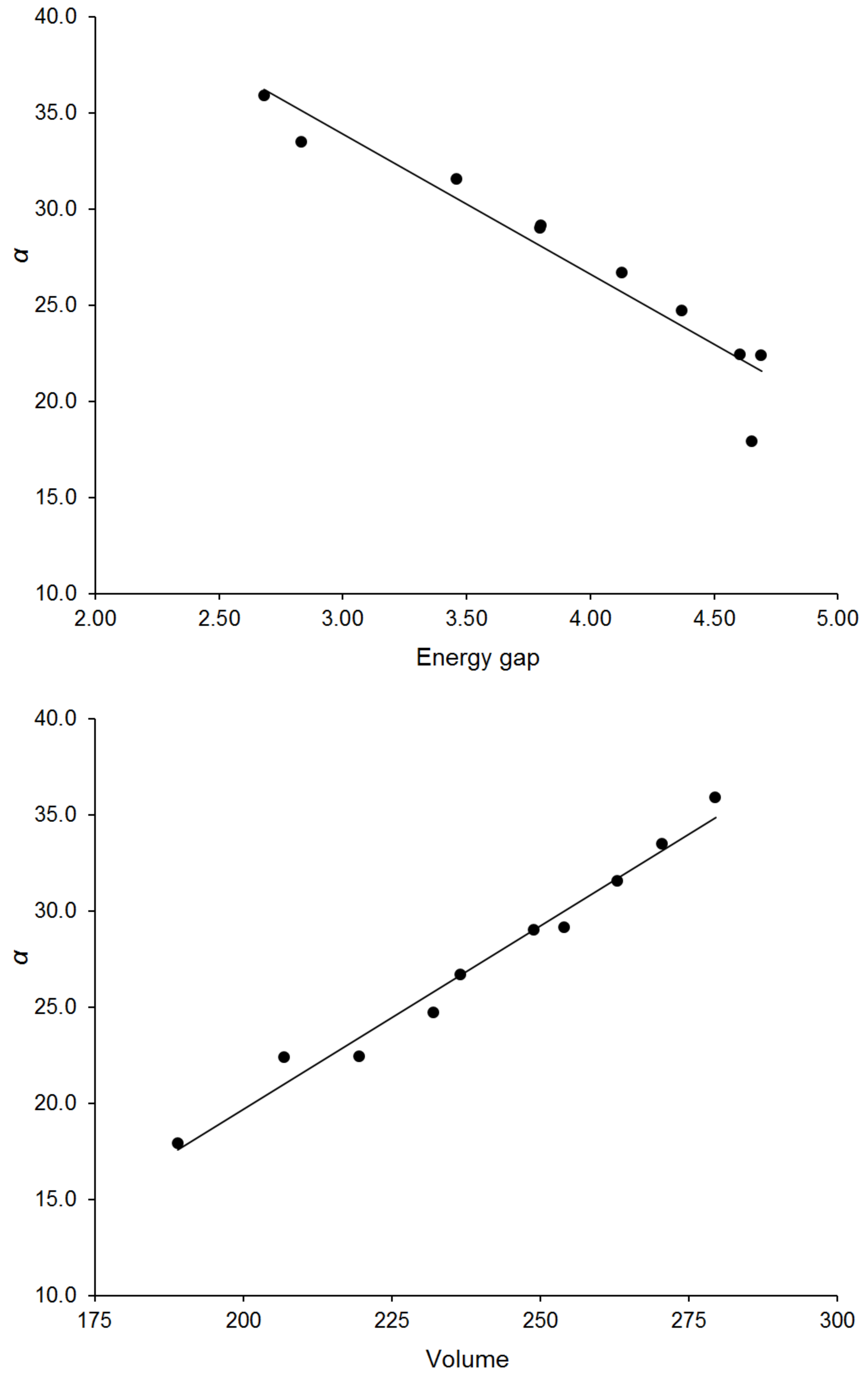


Figure S5. Plots of the polarizability (*α* in Å^3^) against the HOMO-LUMO energy gap (in eV) and the molecular volume (in Å^3^) for the *cc*-conformers of **1a**–**5a** and **1b**–**5b** in vacuum.


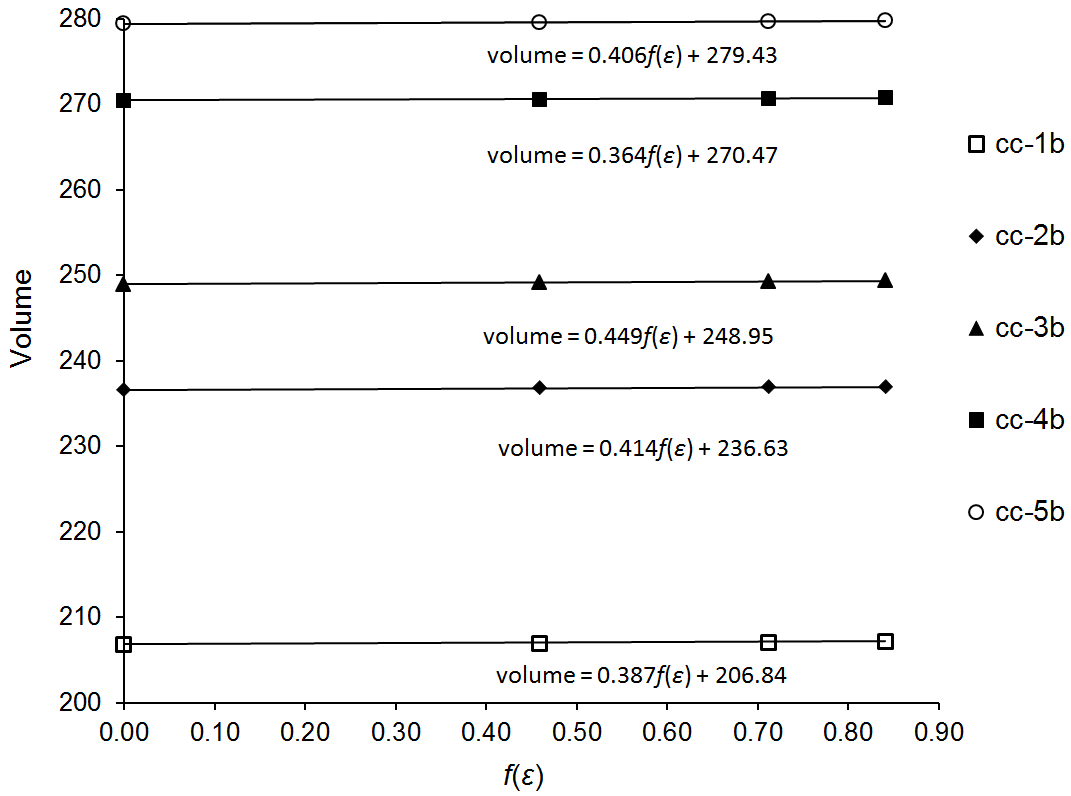


Figure S6. Plot of the molecular volume (in Å^3^) against the COSMO correction factor *f*(*ε*) for the *cc*-conformers of **1b**–**5b** in vacuum and three solvents. The positive slope of the relation between the volume and *f*(*ε*) for each *cc*-conformer indicates the increase of volume with the growing solvent polarity.

**
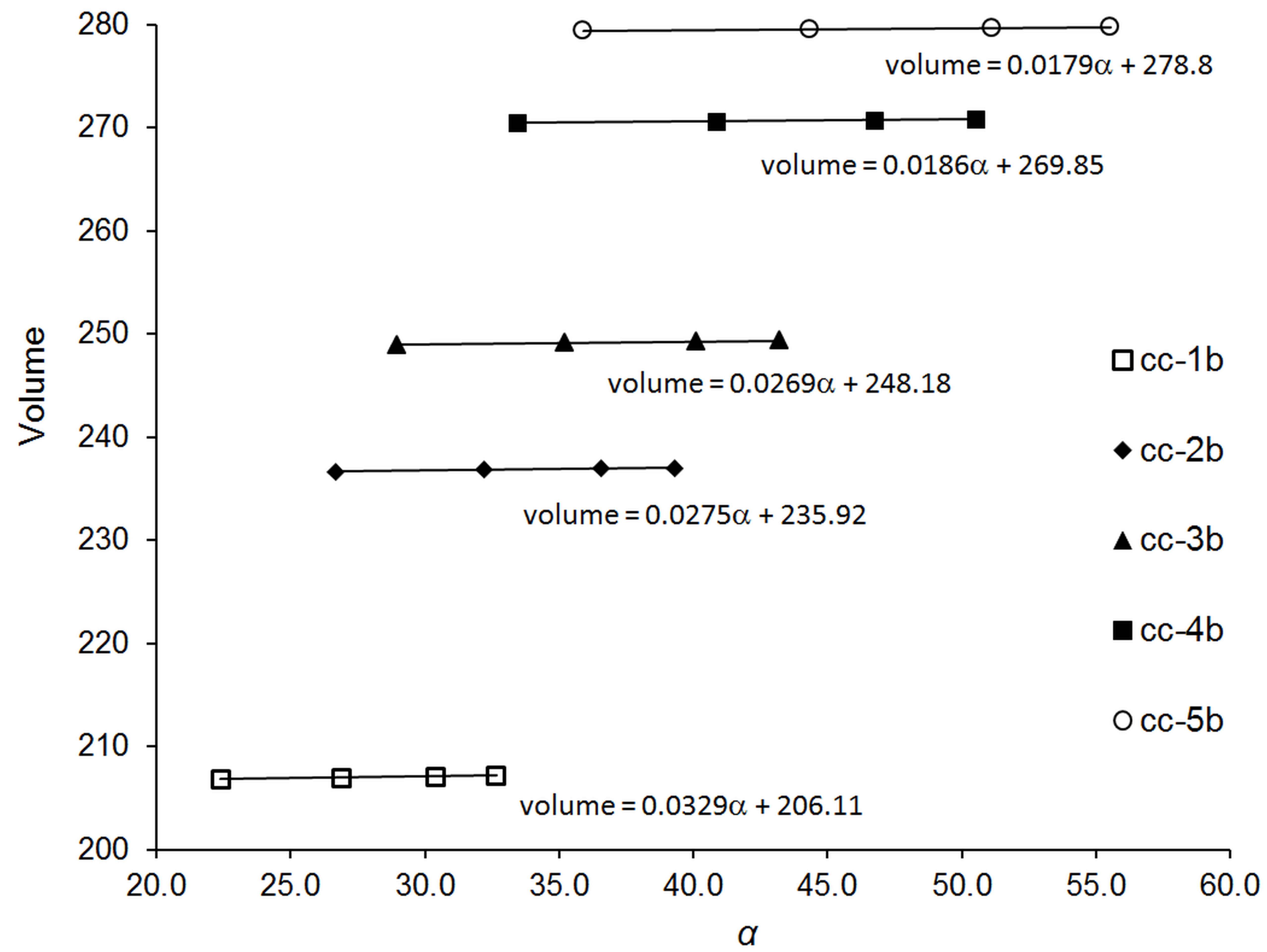
**

Figure S7. Plot of the molecular volume (in Å^3^) against the molecular polarizability (*α* in Å^3^) for the *cc*-conformers of **1b**–**5b** in vacuum and three solvents. The positive slope of the relation between the volume and *α* for each *cc*-conformer indicates that the increase of polarizability is associated with the increase of volume.

**References**

1. Keith TA (2014) AIMAll (Version 14.06.21). TK Gristmill Software, Overland Park KS, USA

2. Hickey AL, Rowley CN (2014) Benchmarking quantum chemical methods for the calculation of molecular dipole moments and polarizabilities. J Phys Chem A 118:3678−3687

3. Nelson Jr. RD, Lide Jr. DR, Maryott AA (1967) Selected Values of Electric Dipole Moments for Molecules in the Gas Phase. NSRDS-NBS10. U.S. Government Printing Office, Washington

4. Hellwege KH, Hellwege AM (1974) Molecular Constants from Microwave, Molecular Beam, and Electron Spin Resonance Spectroscopy, vol 6. Springer-Verlag, Berlin

5. Fringuelli F, Marino G, Taticchi A (1977) Tellurophene and related compounds. Adv Heterocyc Chem 21:119–173

6. Kamada K, Ueda M, Nagao H, Tawa K, Sugino T, Shmizu Y, Ohta K (2000) Molecular design for organic nonlinear optics: polarizability and hyperpolarizabilities of furan homologues investigated by ab initio molecular orbital method. J Phys Chem A 104:4723–4734
